# Supplementary material for: Comprehensive mapping of lunar surface chemistry by adding Chang'e-5 samples with deep learning
Source: Nat Commun. 2023 Nov 20;14:7554. doi: 10.1038/s41467-023-43358-0 (PMC10661975; doi:10.1038/s41467-023-43358-0)
Supplement: Supplementary file 1 — Supplementary Information [file 41467_2023_43358_MOESM1_ESM.pdf]

Supplementary Materials

Comprehensive mapping of lunar surface chemistry by adding Chang'e-5 samples with deep learning

Chen Yang[1,2]*, Xinmei Zhang[1], Lorenzo Bruzzone[3], Bin Liu[2], Dawei Liu[2], Xin Ren[2], Jon Atli Benediktsson[4], Yanchun Liang[5], Bo Yang [5], Minghao Yin[6], Haishi Zhao[5]*, Renchu Guan[5]*, Chunlai Li[2]* & Ziyuan Ouyang [2,7]

[1]College of Earth Sciences, Jilin University, Changchun, China.
[2]Key Laboratory of Lunar and Deep Space Exploration, National Astronomical Observatories, Chinese Academy of Sciences, Beijing, China.
[3]Department of Information Engineering and Computer Science, University of Trento, Trento, Italy.
[4]Faculty of Electrical and Computer Engineering, University of Iceland, 102 Reykjavik, Iceland.
[5]College of Computer Science and Technology, Jilin University, Changchun, China.
[6]College of Information Science and Technology, Northeast Normal University, Changchun, China.
[7]Institute of Geochemistry, Chinese Academy of Sciences, Guiyang, China.

Contacting email: yangc616@jlu.edu.cn; zhaohs@jlu.edu.cn; guanrenchu@jlu.edu.cn; licl@nao.cas.cn

This PDF file includes:

Supplementary Tables 1-3
Supplementary Figures 1-5

Supplementary Table 1 | The measured chemical contents (wt.%) of six major oxides and their MI locations in 55 lunar sampling sites.

Lunar sites		TiO_2	FeO	Al_2O_3	MgO	CaO	SiO_2	MI location	Sample Numbers	References
	A12-1	3.35	14.8	14.95	9.55	10.40	45.95	3°0′46.35″S, 23°25′14.33″W	12003、12070	[1]、[2]、[3]、[4]
	A12-2	3.5	14.3	14.70	9.00	10.70	46.6	3°0′43.74″S, 23°25′22.53″W	12030	[3]
	A12-3	2.9	14.1	15.20	9.40	10.70	46.5	3°1′8.84″S, 23°25′43.81″W	12032	[2]、[3]、[4]
	A12-4	2.70	14	16.20	8.50	11.00	46.2	3°0′43.02″S, 23°25′36.44″W	12033	[3]、[4]、[5]
	A12-5	3.50	14.9	15.10	10.20	10.50	44.8	3°1′10.39″S, 23°25′46.30″W	12037	[1]、[3]、[4]
	A12-6	2.7	14.2	15.40	9.10	10.90	46.8	3°1′13.41″S, 23°25′34.47″W	12041	[3]、[4]
	A12-7	3.2	14.1	15.10	9.80	10.90	46.1	3°1′9.67″S, 23°25′20.39″W	12042	[2]、[3]、[5]
	A12-8	3.6	14.2	14.60	9.50	10.60	46.7	3°1′10.68″S, 23°25′5.76″W	12044	[3]
	A14-LM	1.75	10.53	17.69	9.52	10.32	48.2	3°38′45.40″S, 17°28′18.97″W	14003	[6]
	A14-G	1.68	10.35	17.33	9.58	10.37	48.2	3°38′49.66″S, 17°27′52.39″W	14148,14149,14156	[6]
	A14-Compre	1.7	10.54	17.52	9.41	10.49	48.4	3°38′39.73″S, 17°28′34.13″W	14421	[6]
	A15-LM	1.9	14.38	14.46	9.93	10.47	47.11	26°7′53.29″N, 3°37′58.98″E	15015	[7]
	A15-S1	1.59	15.79	13.15	10.81	10.58	46.73	26°1′51.48″N, 3°36′27.27″E	15071,15081	[8]、[9]
	A15-S2	1.32	11.52	17.52	10.54	11.75	46.4	26°0′40.19″N, 3°35′23.21″E	15091,15101,15211,15221,15231	[8]、[10]、[11]、[12]
	A15-S4	1.55	15.96	13.22	11.19	10.46	46.26	26°1′38.80″N, 3°39′41.19″E	15471	[8]、[9]
	A15-S6	1.5	12.14	16.47	10.51	11.29	46.72	25°58′43.94″N, 3°40′41.53″E	15241,15261,15271,15291	[11]、[12]
	A15-S7	1.25	12.37	15.92	11.17	11.12	46.61	25°59′2.79″N, 3°39′57.19″E	15411,15431	[12]、[13]、[14]
	A15-S8	1.72	14.98	14.46	10.35	10.38	46.94	26°7′59.05″N, 3°37′51.8″E	15013	[15]
	A15-S9	1.78	16.61	12.54	10.98	10.31	46.46	26°7′47.18″N, 3°34′28.4″E	15501	[9]、[16]
	A15-S9a	1.84	20.19	10.31	11.36	9.39	46.52	26°7′45.05″N, 3°33′55.19″E	15531,15601	[8]
	A16-LM	0.6	5.44	26.25	6.25	15.42	45.25	8°58′26.04″S, 15°29′57.86″E	60501,60600,60601	[17]、[18]、[19]
	A16-S1	0.58	5.4	26.60	6.00	15.60	45	8°58′45.17″S, 15°26′59.87″E	61141,61161,61241,61281,61501	[17]、[19]、[20]
	A16-S2	0.6	5.5	27.00	6.05	15.70	44.6	8°58′34.77″S, 15°28′19.61″E	62241,62281	[19]
	A16-S4	0.52	4.6	27.60	5.10	16.20	45.1	9°5′39.88″S, 15°30′32.88″E	64421,64501	[18]、[19]
	A16-S5	0.65	5.85	26.20	6.25	15.00	45.3	9°4′47.99″S, 15°30′38.76″E	65501,65701,65901	[19]、[20]
Apollo	A16-S6	0.67	5.95	26.40	6.25	15.70	45.2	9°4′26.00″S, 15°29′51.36″E	66041, 66081	[19]
	A16-S8	0.56	5.35	26.60	6.30	15.30	45.1	9°4′4.27″S, 15°28′35.61″E	68121,68501,68821,68841	[13]、[19]
	A16-S9	0.61	5.7	26.30	6.30	15.10	45.2	9°3′10.59″S, 15°28′37.36″E	69921, 69941, 69961	[13]、[19]
	A16-S11	0.41	4.2	28.90	4.30	16.50	45.1	8°49′50.31″S, 15°29′35.21″E	67461,67481,67601,67701	[19]
	A16-S13	0.54	4.8	27.60	5.40	15.80	45.1	8°51′15.90″S, 15°30′18.50″E	63321,63341,63501	[13]、[19]
	A17-LM	8.5	16.6	12.10	9.80	11.10	40.8	20°11′19.35″N, 30°46′30.89″E	70011,70181	[21]、[22]、[23]、[24]
	A17-S1	9.6	17.8	10.90	9.60	10.80	39.9	20°9′16.24″N, 30°47′23.30″E	71131,71151,71501	[23]、[24]、[25]
	A17-S2	1.5	8.7	20.70	9.90	12.80	45	20°5′45.86″N, 30°31′49.24″E	72321,72441,72461,72501,72701	[22]、[23]、[24]、[25]、[26]
	A17-S3	1.8	8.7	20.40	10.20	12.90	44.9	20°10′10.33″N, 30°33′59.49″E	73211,73221,73261,73281	[21]、[22]、[24]
	A17-S5	9.9	17.7	10.90	9.60	10.80	39.8	20°11′1.25″N, 30°43′43.39″E	75061,75081	[22]、[23]、[25]、[26]
	A17-S6	3.4	10.7	18.30	10.80	12.20	43.5	20°17′18.36″N, 30°48′14.88″E	76221,76241,76261,76501	[22]、[23]、[24]
	A17-S7	3.9	11.6	17.30	10.10	11.90	43.7	20°17′21.19″N, 30°49′19.22″E	77511,77531	[23]、[24]
	A17-S8	4.3	12.3	16.60	10.20	11.80	43.4	20°16′31.26″N, 30°52′57.17″E	78221,78231,78441,78461	[24]、[26]
	A17-S9	6.4	15.4	13.90	10.00	11.30	42.1	20°13′48.79″N, 30°50′12.11″E	79211,79511	[22]、[23]、[24]、[25]
	A17-LRV1	8	16.3	12.60	9.40	11.20	41.3	20°10′21.97″N, 30°41′25.36″E	72131	[24]
	A17-LRV2	4.4	13.4	16.10	10.30	11.90	43.1	20°10′43.41″N, 30°38′54.60″E	72141	[21]、[23]
	A17-LRV3	5.5	14.8	14.40	10.40	11.30	42.2	20°10′51.04″N, 30°37′44.49″E	72151,72161	[23]、[24]
	A17-LRV4	1.3	8.5	21.40	9.60	12.80	44.9	20°6′26.50″N, 30°32′55.69″E	73151	[24]
	A17-LRV5	2.6	9.8	19.90	8.90	12.80	44.8	20°10′51.77″N, 30°35′35.69″E	74111	[24]
	A17-LRV6	2.6	10.3	19.40	9.90	12.50	44.5	20°11′31.13″N, 30°36′0.89″E	74121	[21]、[26]
	A17-LRV7	6.8	16.1	12.80	10.30	10.70	41.8	20°12′46.46″N, 30°40′14.71″E	75111	[24]
	A17-LRV8	6.6	15.7	13.50	9.90	11.30	41.9	20°12′23.07″N, 30°41′42.64″E	75121	[24]
	A17-LRV9	6.1	14.6	14.30	9.80	11.30	42.2	20°13′56.19″N, 30°47′11.17″E	76121	[24]
	A17-LRV10	3.7	11.2	17.50	10.50	12.10	43.5	20°16′48.31″N, 30°47′37.59″E	76131	[24]
	A17-LRV11	4.5	12.7	16.30	10.00	11.90	43.2	20°16′21.57″N, 30°52′32.96″E	78121	[24]
	A17-LRV12	10	17.4	11.20	9.36	10.80	39.9	20°11′41.55″N, 30°48′58.56″E	70311,70321	[24]
	L16	3.41	16.85	15.24	8.67	12.38	41.5	0°30′53.84″S, 56°21′52.57″E	luna16s	[27]、[28]
Luna	L20	0.46	7.5	22.90	9.15	14.50	45.6	3°47′10.84″N, 56°37′27.00″E	luna20s	[19]
	L24	1.1	20.5	11.10	10.20	11.00	45.4	12°42′51.11″N, 62°12′49.79″E	24077,24109,24149,24174,24182,24210	[29]
Chang'e	CE-5	5	22.5	10.80	6.48	11.00	42.2	43°3′28.80″N, 51°54′57.60″W	CE-5	[30]

The chemical contents presented here are the average values of the measured lunar surface chemical abundances from the returned samples. References: [1]Wanke *et al.*,(1971); [2]Morrison *et al.*,(1971); [3]Frondel *et al.*,(1971); [4]Korotev and Rockow(1995); [5]Cuttitm *et al.*,(1971); [6]Philpotts *et al.*,(1972); [7]Scoon *et al.*,(1977); [8]Wanke *et al.*,(1973); [9]Duncan *et al.*,(1975); [10]Carron *et al.*,(1972); [11]Cuttitta *et al.*,(1973); [12]Korotev(1987a); [13]Willis *et al.*,(1972); [14]Rose *et al.*,(1975); [15]Christian *et al.*,(1976); [16]Apollo 15 Preliminary Examination Team(1972); [17]Rose *et al.*,(1973); [18]Apollo 16 Preliminary Examination Team(1973); [19]Korotev(1981); [20]Korotev(1982); [21]Wanke *et al.*,(1974); [22]Rose *et al.*,(1974); [23] Rhodes *et al.*,(1974); [24]Korotev and Kremser(1992); [25]Laul *et al.*,(1974); [26]Duncan *et al.*,(1974); [27]Gillurn *et al.*,(1972); [28]Vinogradov(1971); [29] Blanchard *et al.*,(1978); [30]Li *et al.*,(2021).

Supplementary Table 2 | The accuracies (RMSEs) of inversion results from the existing sampling points.

Data	Methods	TiO_2	FeO	Al_2O_3	MgO	CaO	SiO_2
Clementine UVVIS	Lucey et al. [5]	1.3076	0.9047	-	-	-	-
LP GRS	Prettyman et al. [35]	1.8915	4.0542	5.4899	-	-	-
Chang'e-1 IIM	Xia et al. [9]	0.3156	**0.1993**	0.4020	0.1521	0.3839	0.5495
Diviner CF	Ma et al. [22]	2.0293	2.4107	2.1391	0.7206	0.9925	1.9851
	Wang et al. [24]	0.9861	1.1486	0.9866	0.4337	0.7299	-
SELENE MI	Zhang et al. [23]	0.2617	0.3576	0.4447	0.2193	0.3814	-
	This work	**0.2168**	0.2020	**0.2968**	**0.1254**	**0.1259**	**0.1724**

For abundance algorithms by Lucey et al., only TiO_2 and FeO abundances were derived. In inversion methods with SELENE MI by Wang et al. and Zhang et al., five oxides abundances were estimated. The sampling points applied in different literatures of Clementine UVVIS, Diviner CF, Chang'e-1 IIM, SELENE MI (Wang et al. and Zhang et al.) are used for calculating RMSEs. This work and LP GRS use the same sample points. All the measured and predicted values can be found in Supplementary Fig. 4.

Supplementary Table 3 | The performance (RMSEs and R^2) of the designed 1D CNN inversion model and the compared ELM and GOP methods under the LOOCV configuration.

Methods	TiO_2		FeO		Al_2O_3		MgO		CaO		SiO_2	
	RMSE	R^2	RMSE	R^2	RMSE	R^2	RMSE	R^2	RMSE	R^2	RMSE	R^2
ELM [64]	1.0411	0.8348	1.0103	0.9488	1.3146	0.9366	0.86	0.7576	0.6102	0.8956	1.0303	0.7721
GOP [65]	1.6472	0.5864	2.2764	0.7400	1.8158	0.8790	0.8914	0.7395	0.8938	0.7761	0.9937	0.7880
This work	**0.3889**	**0.9769**	**0.8011**	**0.9678**	**0.6471**	**0.9846**	**0.4659**	**0.9289**	**0.3680**	**0.9620**	**0.4232**	**0.9616**

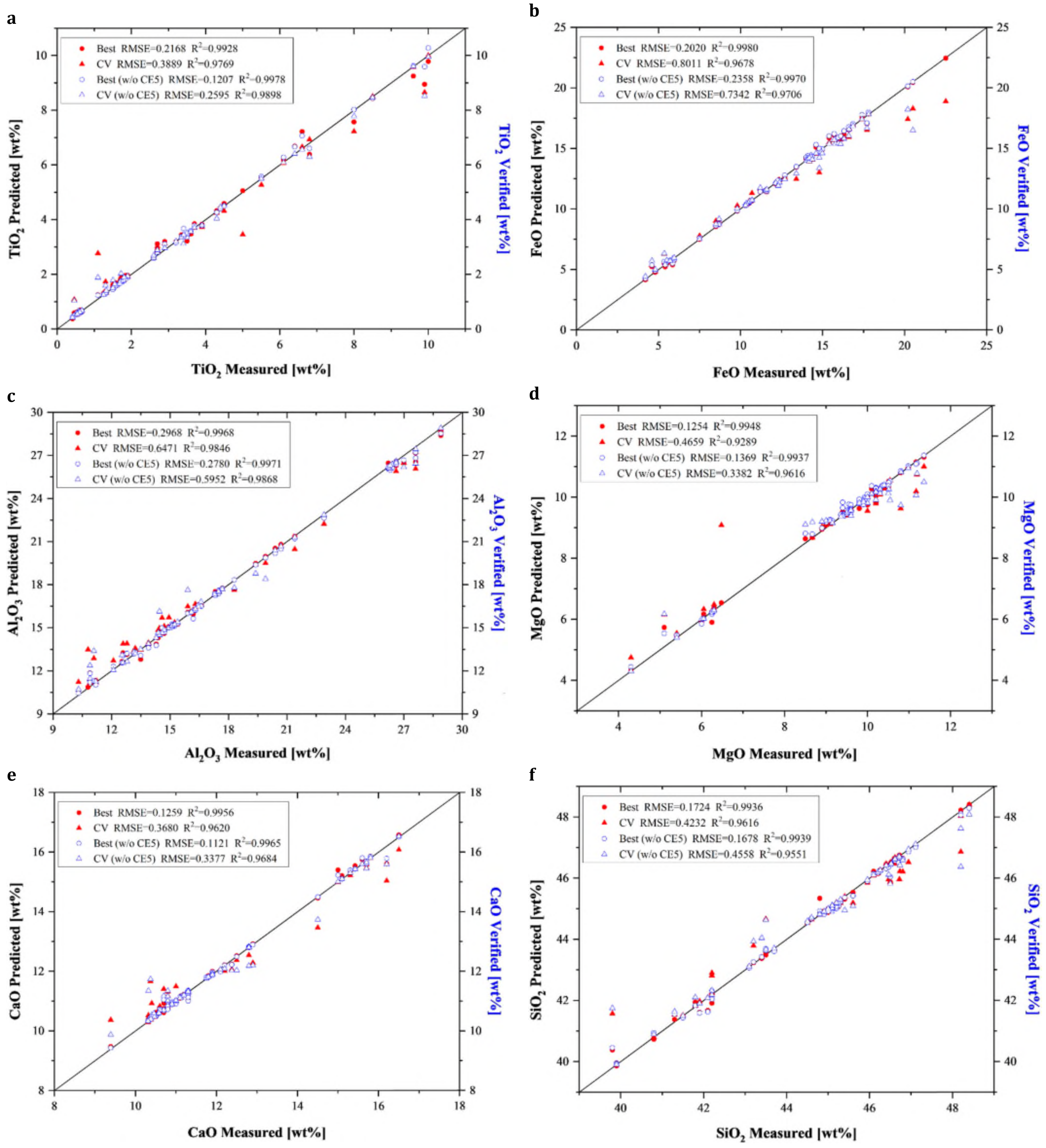

Supplementary Fig. 1 | Prediction and validation accuracies of the deep learning-based inversion model. **a**, **b**, **c**, **d**, **e**, **f** and **g** show the accuracy evaluation of the six major oxides TiO_2, FeO, Al_2O_3, MgO, CaO and SiO_2, respectively. For the 55 sampling points from Apollo, Luna and Chang'e-5 mission, the red dots and triangles indicate the best prediction precisions by the 1D convolutional neural network-based inversion algorithm and the validation accuracies by leave-one-out-cross-validation. The blue dots and triangles are the best prediction and validation results without Chang'e-5 sample.

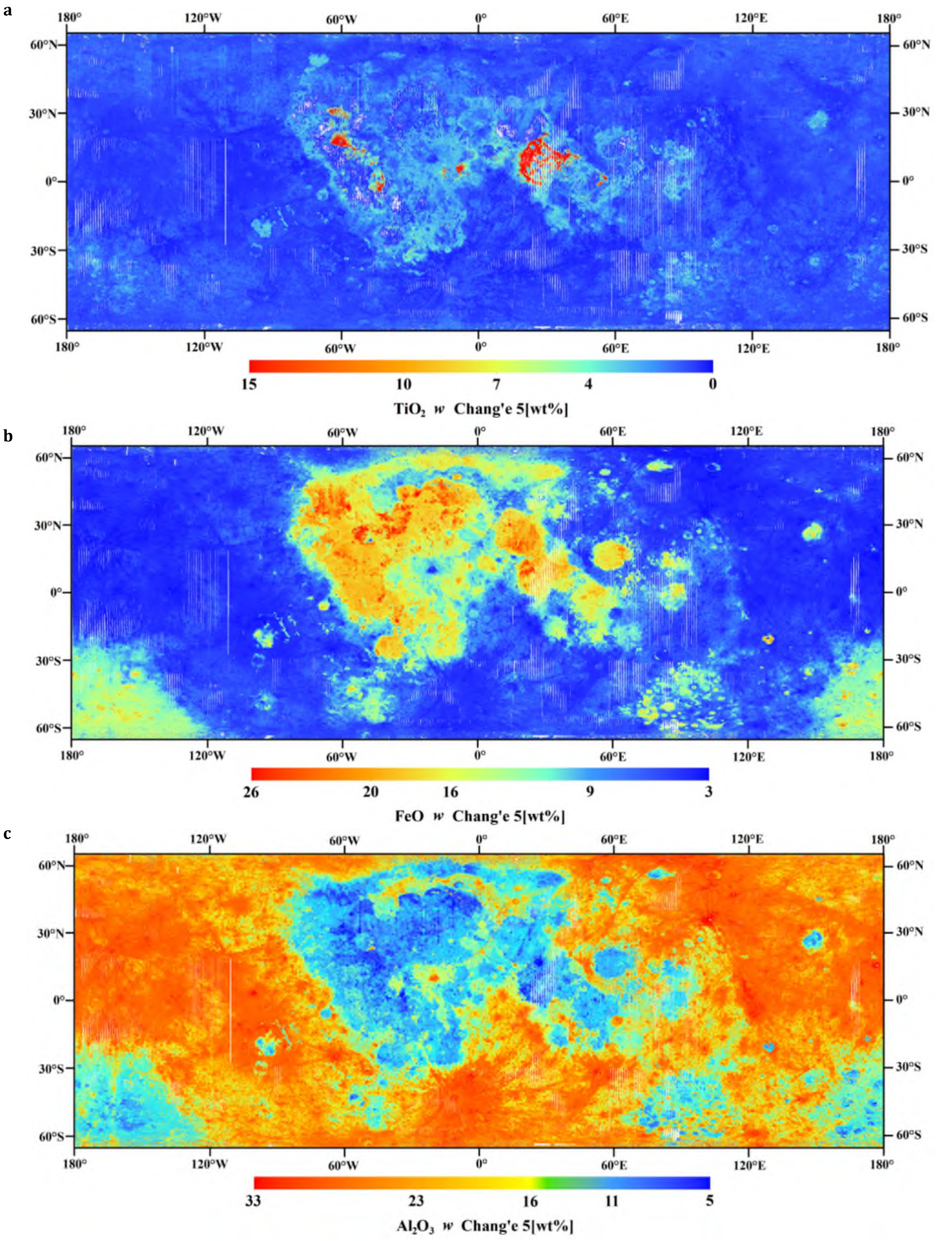

Supplementary Fig. 2 | The lunar surface chemistry maps of six major oxides only with Apollo and Luna data (w/o CE5). a, **b** and **c** show the maps of TiO_2, FeO and Al_2O_3 abundances calculated from the deep learning-based inversion method, respectively.

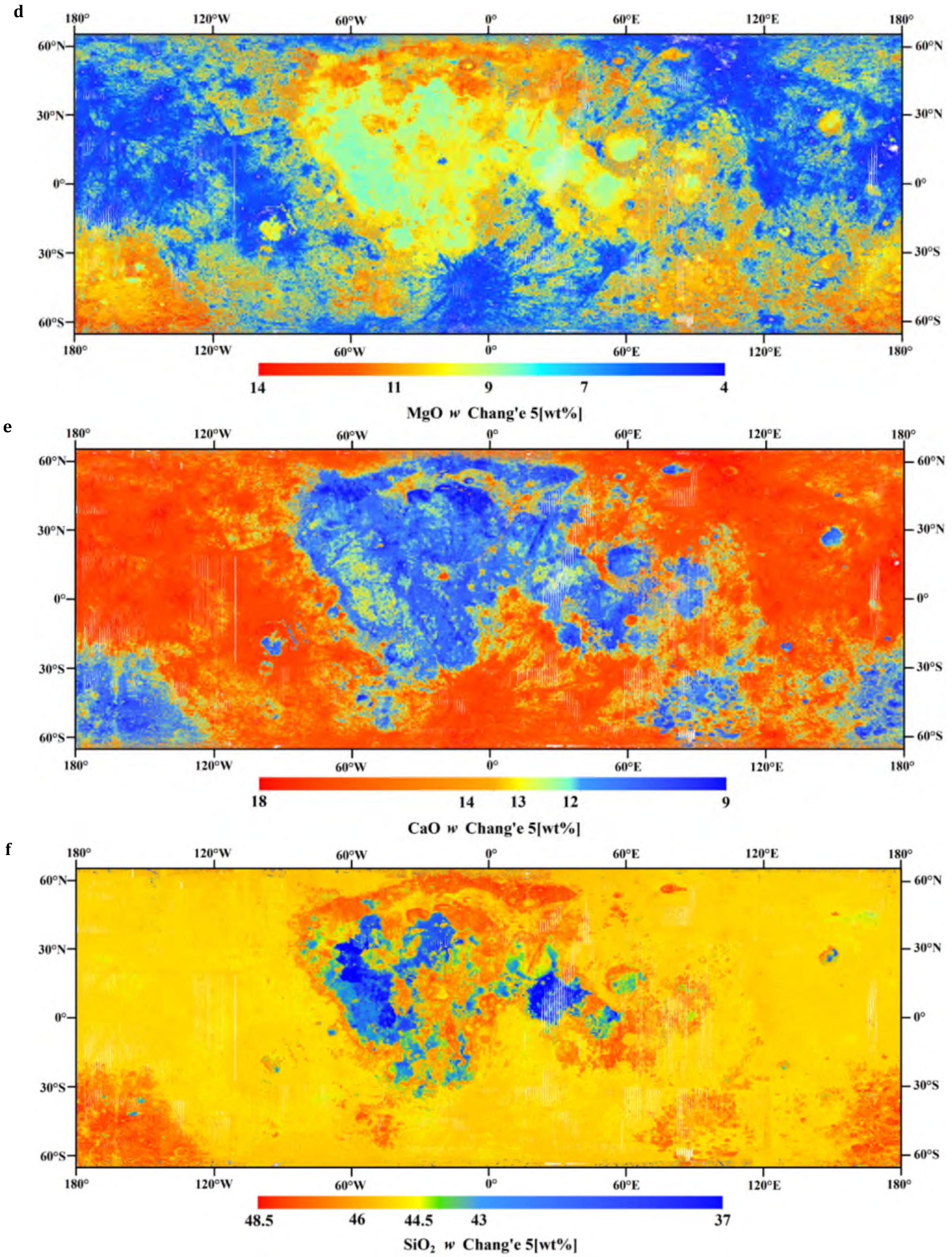

Supplementary Fig. 2 | The lunar surface chemistry maps of six major oxides only with Apollo and Luna data (w/o CE5) (*continued*). d, **e** and **f** show the maps of MgO, CaO and SiO_2 abundances calculated from the deep learning-based inversion method, respectively.

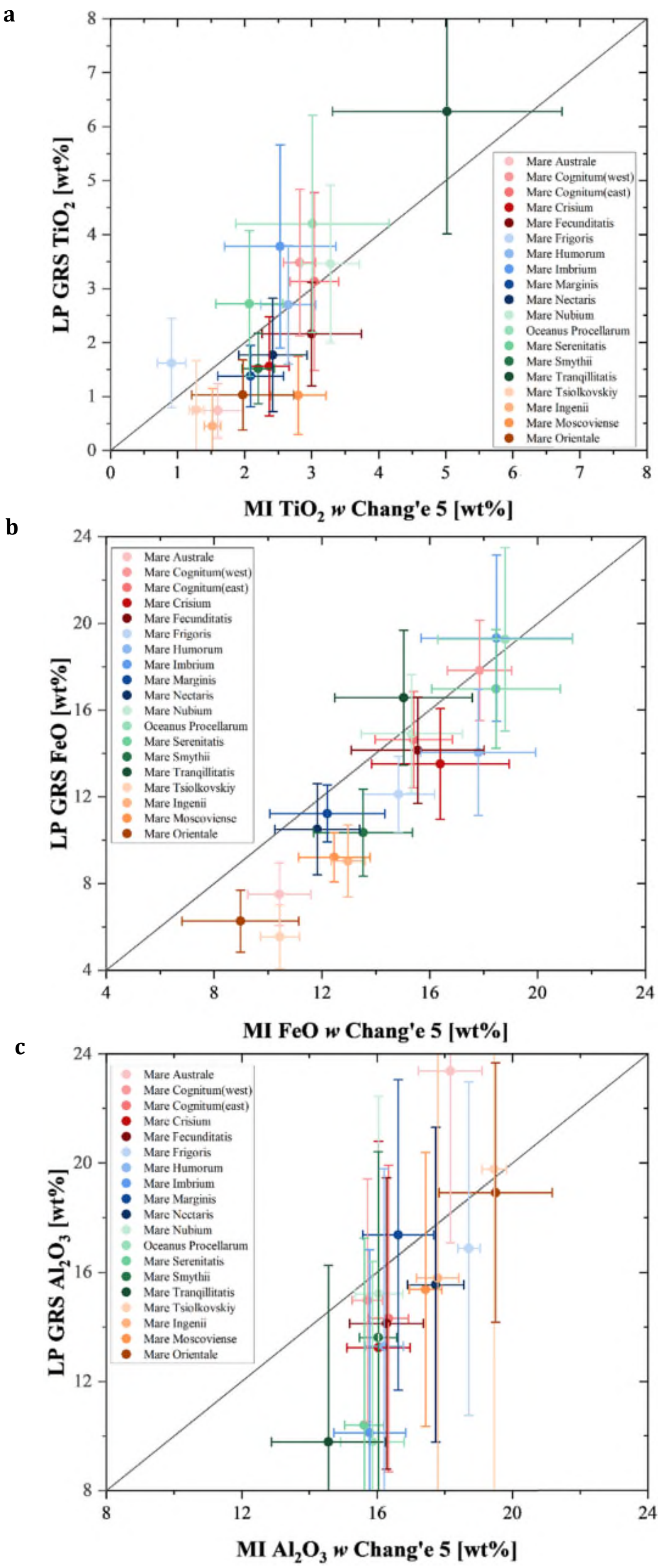

Supplementary Fig. 3 | The contents of the six major oxides from Lunar Prospector (LP) Gamma-Ray Spectrometer (GRS) maps and SELENE (KAGUYA) multiband imager (MI) data by adding Chang'e 5 samples for lunar mare. a, **b** and **c** show the averaged TiO_2, FeO and Al_2O_3 contents, respectively. Error bar indicates the standard deviation. Solid line is the 1:1 line. The Chang'e-5 inversion maps were down-sampled to the LP GR resolution, i.e., 2° per pixel.

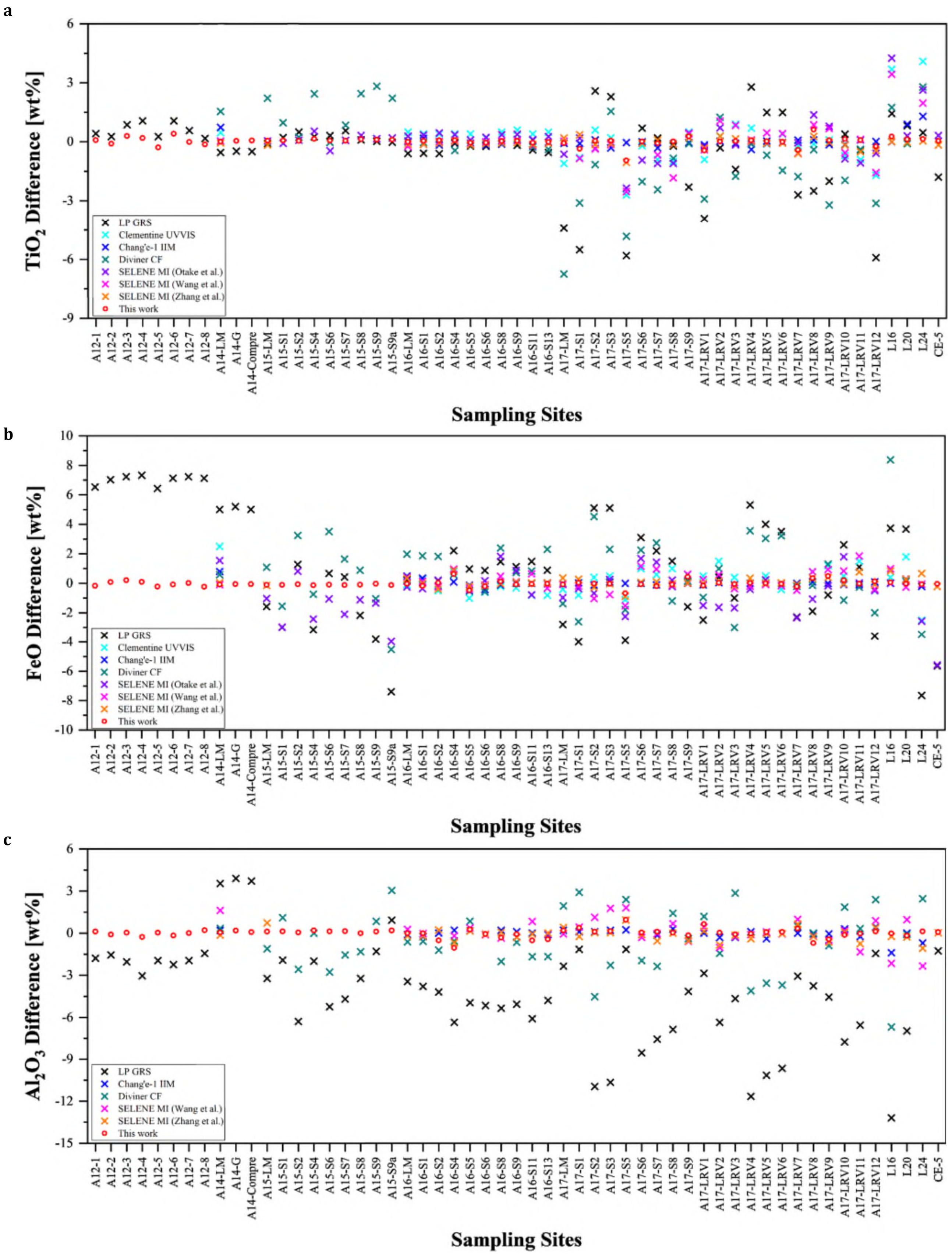

Supplementary Fig. 4 | Scatter diagram of differences between measured abundances and estimated values of major oxides in the sampling sites. a, **b** and **c** show the differences of major oxides, i.e., TiO_2, FeO and Al_2O_3, calculated from the sample compositional data and the abundance algorithms or inversion methods, respectively. The Apollo 11 results were not extracted from the Chang'e-1 IIM images and Apollo 15 values were not involved in the SELENE MI for data missing.

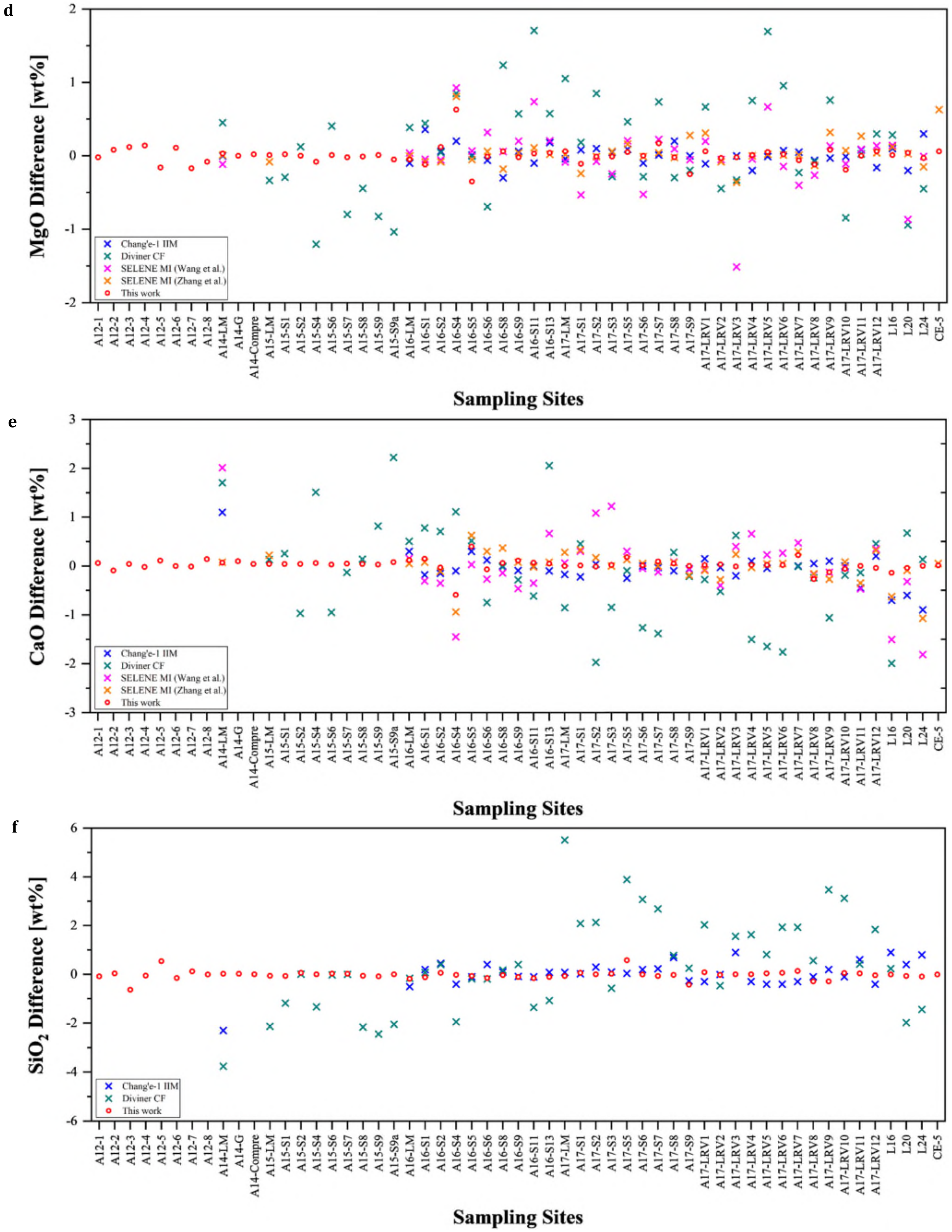

Supplementary Fig. 4 | Scatter diagram of differences between measured abundances and estimated values of major oxides in the sampling sites (*continued*). d, e and f show the differences of major oxides, i.e., MgO, CaO and SiO_2 calculated from the sample compositional data and the abundance algorithms or inversion methods, respectively. The Apollo 11 results were not extracted from the Chang'e-1 IIM images and Apollo 15 values were not involved in the SELENE MI for data missing.

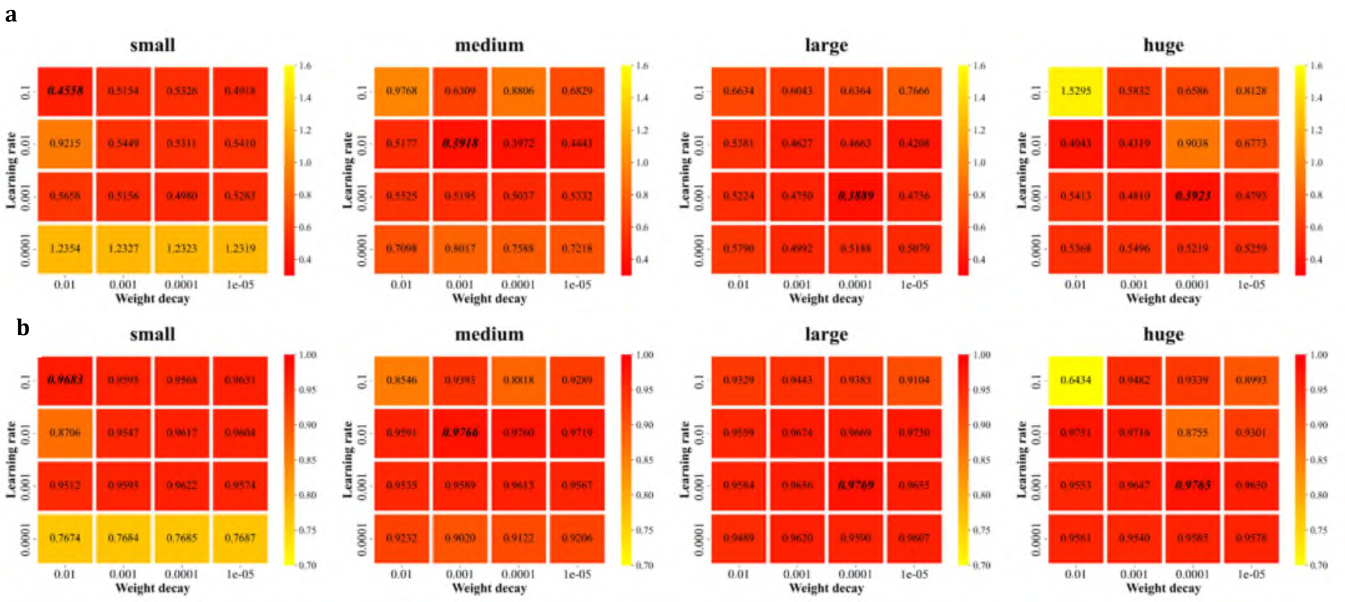

Supplementary Fig. 5 | Ablation experimental results of the 1D CNN inversion model with different model size, learning rate and weight decay on TiO_2. **a** shows the RMSE results under the LOOCV setup, where the four subplots represent the ablation experiment results at all combinations of learning rates and weight decay for the 'small', 'medium', 'large' and 'huge' models, in which smaller RMSE values indicate better performance. The best RMSE is bolded and italicized. **b** shows the R^2 results with the same settings as **a**, except that smaller R^2 values indicate better performance. The best R^2 is bolded and italicized.
