# Supplementary material for: Comprehensive mapping of lunar surface chemistry by adding Chang'e-5 samples with deep learning
Source: Nat Commun. 2023 Nov 20;14:7554. doi: 10.1038/s41467-023-43358-0 (PMC10661975; doi:10.1038/s41467-023-43358-0)
Supplement: Supplementary file 3 — Description of Additional Supplementary Files [file 41467_2023_43358_MOESM3_ESM.pdf]

Description of Additional Supplementary Files

File Name: Supplementary Data 1
Description: The measured chemical contents with their MI spectral data in lunar sampling sites.

File Name: Supplementary Data 2
Description: Ablation experiment results of the 1D CNN inversion model with different model size, learning rate and weight decay on oxides FeO, Al_2O_3, MgO, CaO and SiO_2.

File Name: Supplementary Data 3
Description: Three Lunar and Young Mare Basalt Geological Boundaries.
