# Supplementary material for: Comprehensive mapping of lunar surface chemistry by adding Chang'e-5 samples with deep learning
Source: Nat Commun. 2023 Nov 20;14:7554. doi: 10.1038/s41467-023-43358-0 (PMC10661975; doi:10.1038/s41467-023-43358-0)
Supplement: Supplementary file 5 — Supplementary Data 2 [file 41467_2023_43358_MOESM5_ESM.docx]

**Supplementary Data 2: Ablation experiment results of the 1D CNN inversion model with different model size, learning rate and weight decay on oxides FeO, Al_2_O_3_, MgO, CaO and SiO_2_.**

**FeO：**

| 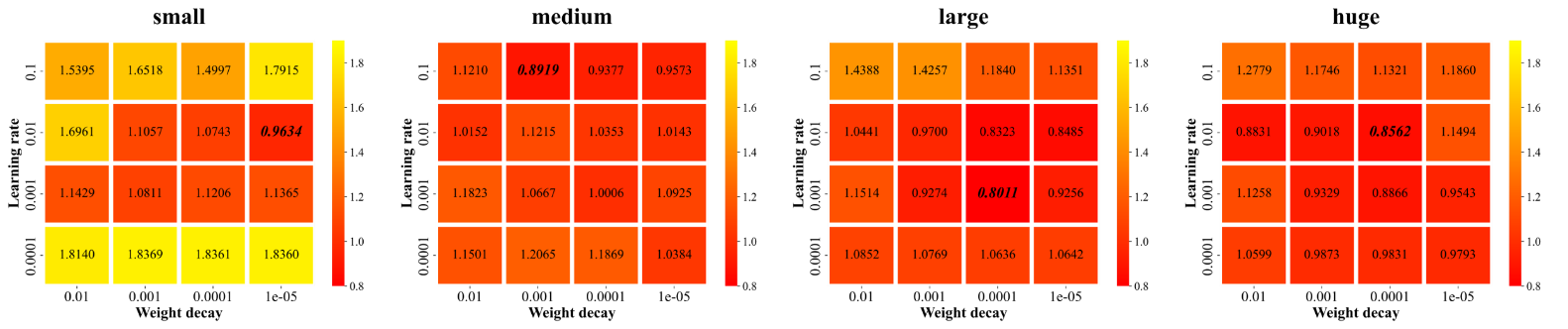  **a** |
| --- |
| 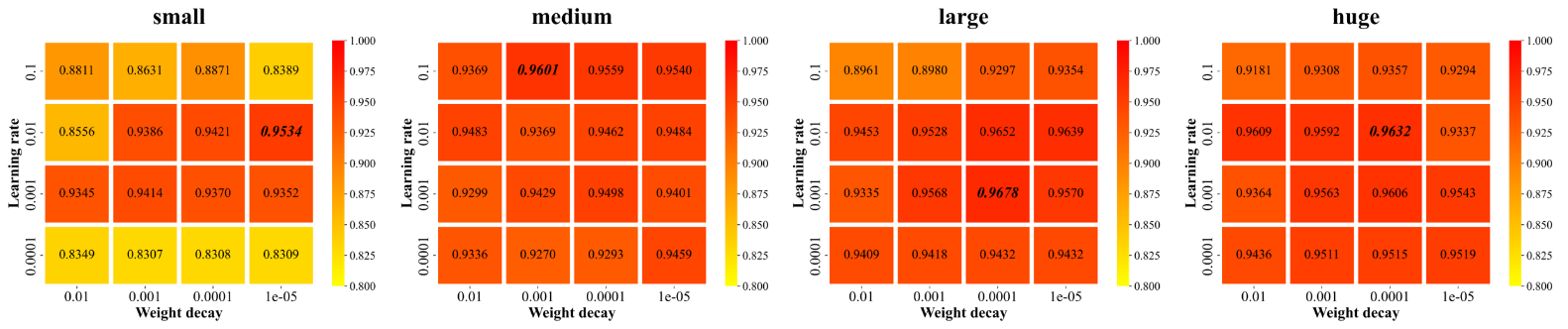  **b** |

**Al_2_O_3_：**

| 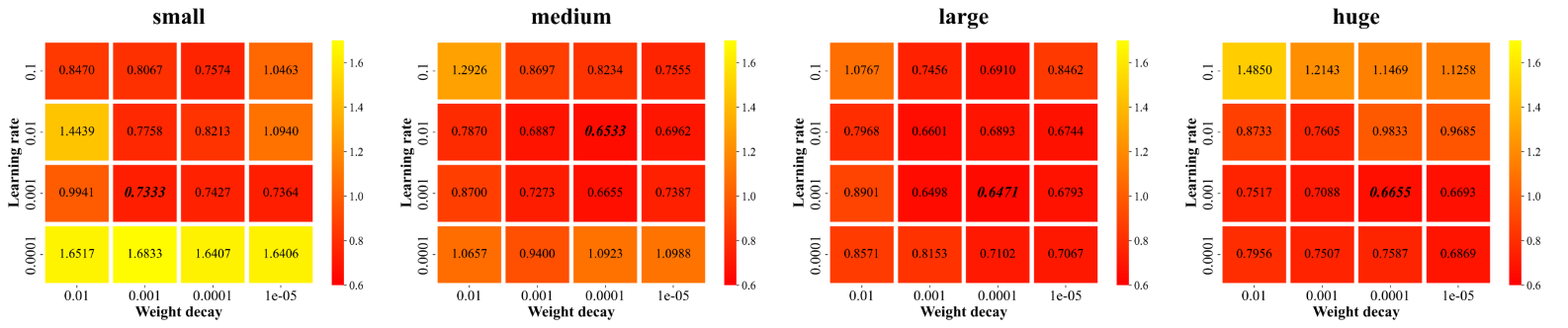  **a** |
| --- |
| 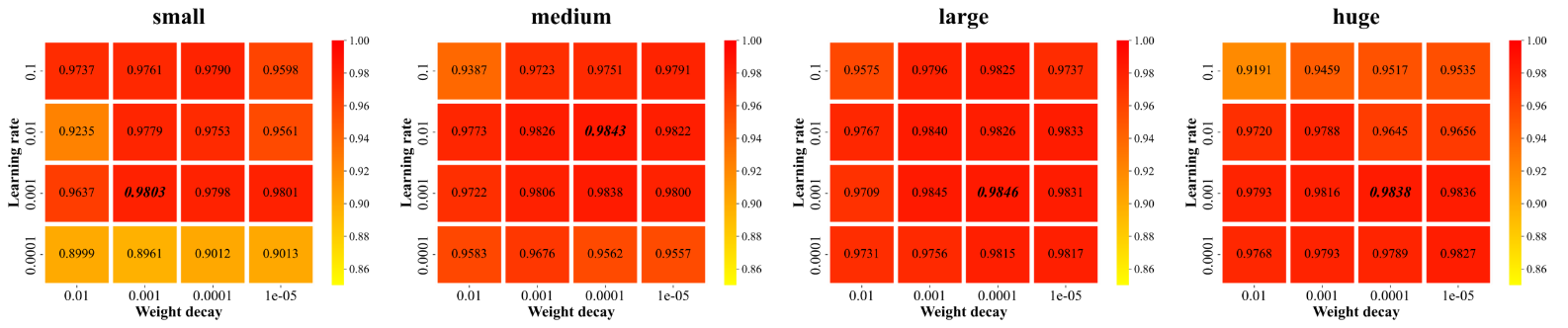  **b** |

**MgO：**

| 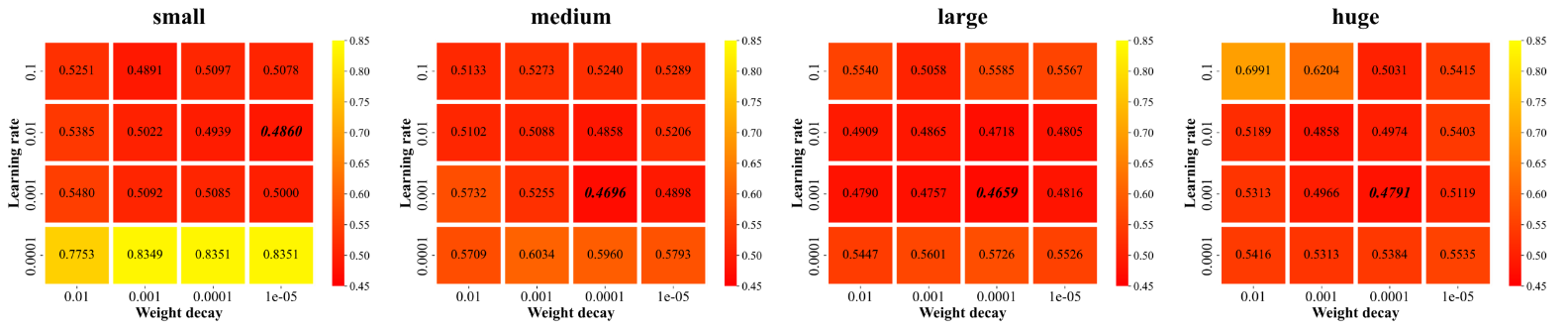  **a** |
| --- |
| 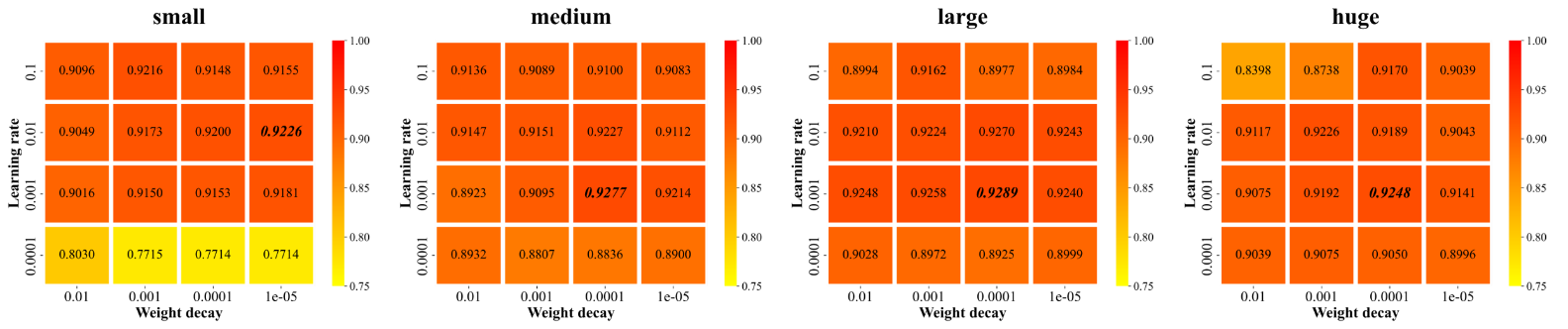  **b** |

**CaO：**

| 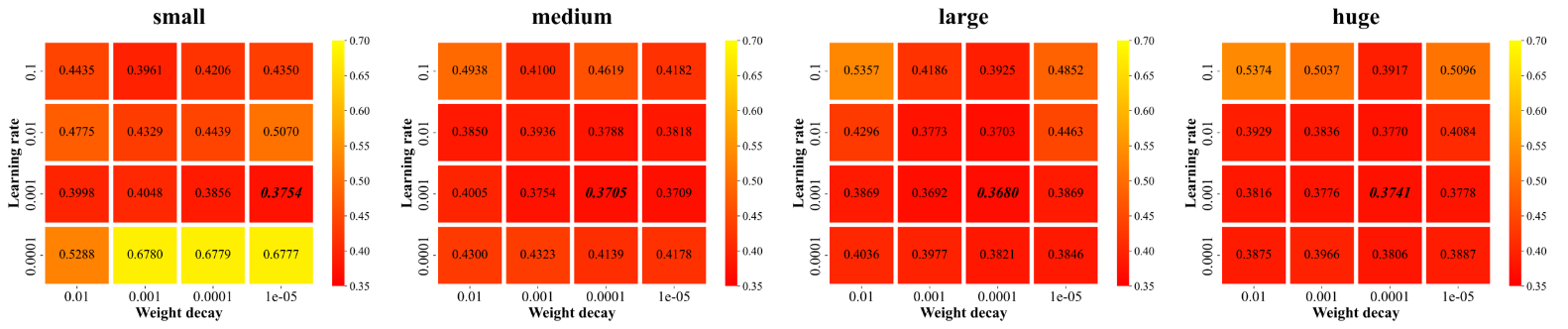  **a** |
| --- |
| 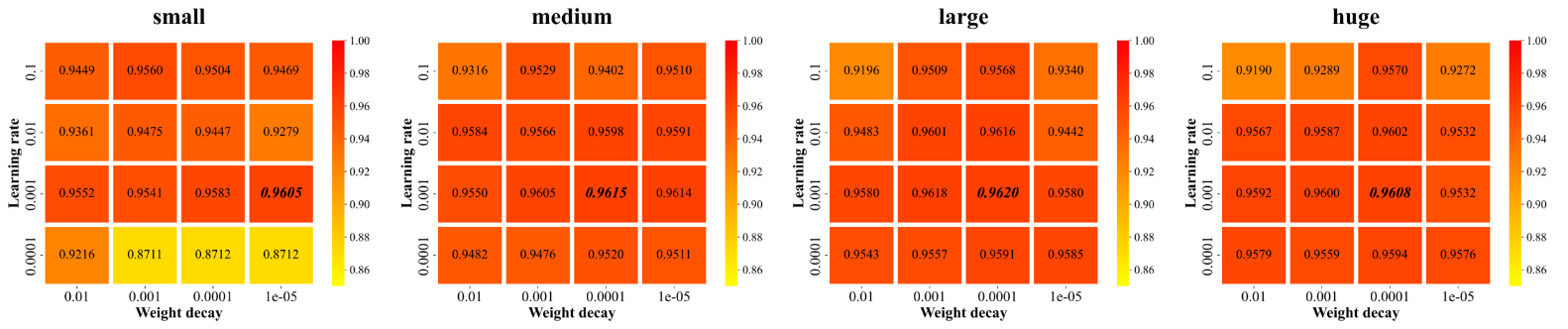  **b** |

**SiO_2_：**

| 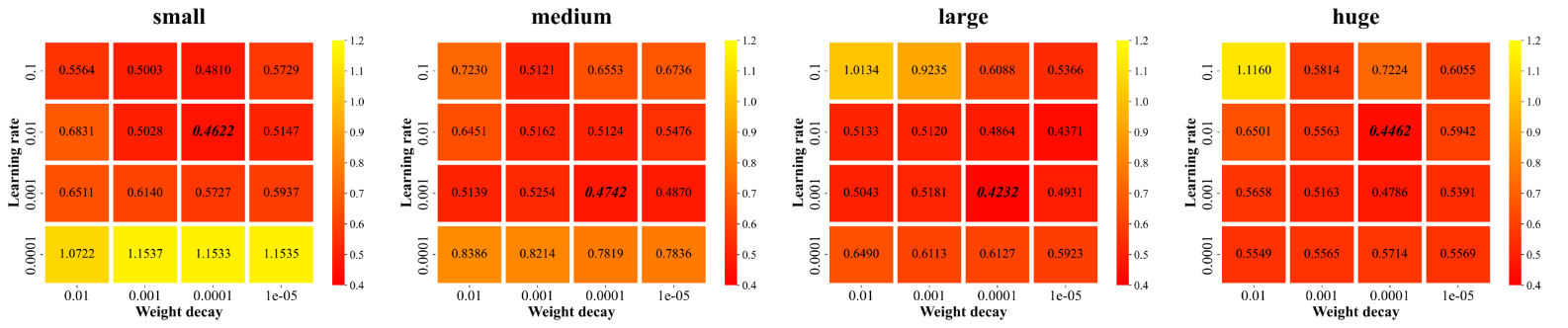  **a** |
| --- |
| 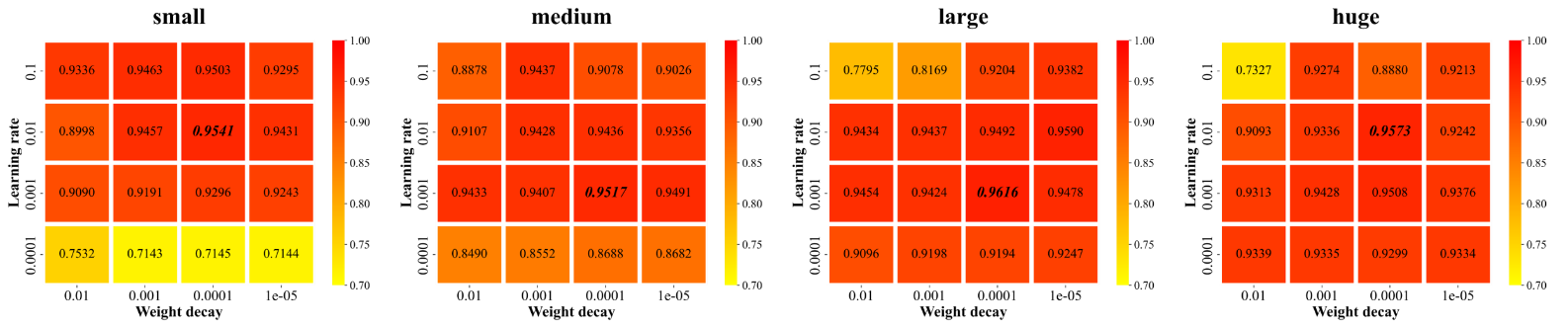  **b** |
